# A. V. M. A.

**Published:** 1901-09

**Authors:** 


### A. V. M. A.

Dr. J. O. Roberts, of Greenville, Mississippi, will be a delegate and visitor at Atlantic City in September.

Dr. Frederick P. Ruhl, Resident State Secretary of West Virginia, will be in attendance at the Atlantic City meeting.

Dr. W. C. Fair, of Cleveland, Ohio, will be among the visitors at the Atlantic City meeting. Dr. Fair is editor of the Ohio *Farmer* and Michigan *Farmer*.

Dr. C. A. Cary's paper will be entitled "Some Obstructions in the Way of Efficient Meat-inspection and Milk-inspection."

Papers will also be presented by Dr. L. Van Es, of Mobile, Ala., and Dr. J. C. Robert, Agricultural College, Miss.

Dr. James A. Waugh, of Pittsburg, Pa., will be among those in attendance at Atlantic City in September.

Mrs. G. E. Nesom, of Clemson College, S. C., will be among the visitors at Atlantic City.

Senator Kenney will be among the invited guests at the banquet of the Association at the "Rudolf" on Thursday evening, September 5, 1901.

Hon. Franklin P. Stoy, Mayor of Atlantic City, will welcome the members and delegates at the opening session of the convention and contribute to their pleasure and comfort during their sojourn at the famous seaside resort.

